# Factors affecting occupational therapy services for patients with traumatic brain injury

**DOI:** 10.4102/ajod.v12i0.1203

**Published:** 2023-12-06

**Authors:** Melanie C.J.S. Leyder, Fiona Breytenbach

**Affiliations:** 1 Department of Occupational Therapy, Faculty of Health Sciences, University of the Witwatersrand, Johannesburg, South Africa

**Keywords:** traumatic brain injury, occupational therapy, rehabilitation, barriers, facilitators, focus group, Gauteng

## Abstract

**Background:**

Traumatic brain injury (TBI) is one of the top causes of disability in the younger population worldwide. Rehabilitation services should be accessible to all citizens to achieve universal health coverage.

**Objectives:**

This study aimed to explore the barriers and facilitators influencing the provision of rehabilitation for patients with TBI from occupational therapists’ perspectives in Gauteng, South African healthcare facilities. It further explored the perceived strategies that would strengthen the delivery and provision of TBI rehabilitation.

**Method:**

An exploratory qualitative research design was used in this study. A total of 16 occupational therapists were in focus groups either face-to-face or on an online platform. Thematic analysis was used to analyse the raw data.

**Results:**

There were three themes that emerged from the study, namely, ‘Not a recipe’, ‘Toolbox’, and ‘Lost in the cracks’. These themes highlighted the various aspects affecting the rehabilitation of patients with TBI.

**Conclusion:**

There are factors that both enhance and hinder service delivery for patients with TBI rehabilitation. Strategies to overcome barriers to TBI rehabilitation need to be considered to improve service provision for patients with TBI.

**Contribution:**

This article provides recommendations to improve occupational therapy services for patients with TBI in South Africa. The findings can contribute to policy development and education requirements for TBI.

## Introduction

Traumatic brain injury (TBI) is one of the leading causes of disability in low- and middle-income countries (LMICs) (Wong et al. 2016). The growing urbanisation of African cities brings an anticipated increase of motor vehicle accidents and a predicted 6 to 14 million new cases in Africa by the year 2050 (Wong et al. 2016). The expected rise in TBI warrants an urgent call to train healthcare professionals in Africa to lessen mortality and morbidity rates because of TBI. While neurointensive care seeks to improve survival rates of TBI cases, rehabilitation mitigates the social and economic burdens of disability because of TBI. The World Health Organization (WHO) defines rehabilitation as a ‘set of interventions designed to optimize functioning and reduce disability in individuals with health conditions in interaction with their environment’ (WHO 2017:35). The WHO’s Rehabilitation 2030 initiative advocates for rehabilitation as an essential health service which should be accessible to all persons to achieve universal health coverage (WHO 2017:3). Furthermore, rehabilitation should be administered by a team due the broad scope of specialisations needed to treat complex health conditions such as TBI (WHO 2017:3). This rehabilitation team ideally includes physiotherapy, physical and rehabilitation medicine, prosthetics and orthotics, psychology, social work, speech and language therapy, and occupational therapy (WHO 2017:15). Occupational therapists form a key part of the rehabilitation team and specifically aim to restore an individual’s independence in daily living including self-care tasks, productivity, as well as home and community integration to lessen the burdens of disability (WHO 2017:14).

Factors influencing TBI rehabilitation have been explored in high-income countries among the rehabilitation team. Patient’s personal factors, environmental influences, the therapeutic relationship and TBI sequelae are perceived factors contributing to the complexity of TBI rehabilitation (Jeyaraj et al. 2013; Lam Wai Shun et al. 2017). Facilitators to TBI outpatient rehabilitation include a well co-ordinated multidisciplinary team (MDT) and quality therapeutic intervention (Jeyaraj et al. 2013). Perceived barriers to TBI outpatient rehabilitation include a lack of resources, limited training and decreased patient time (Jeyaraj et al. 2013). Additional factors affecting individual specialisations or professions have been found. Lam Wai Shun et al. (2017) reported on factors perceived by occupational therapists regarding the rehabilitation potential of TBI patients in Australia. These factors include organisational context, professional expertise, knowledge of scientific evidence and decision-making processes (Lam Wai Shun et al. 2017). However, no literature on the experiences of occupational therapists in LMICs with regard to TBI rehabilitation was found. The aim of this study was to explore the barriers and facilitators to the provision of TBI rehabilitation from occupational therapists’ perspectives in public healthcare settings in the Gauteng province of South Africa. The study further explored the potential strategies to strengthen occupational therapy service delivery for patients with TBI.

## Research methods and design

### Study design

The study used an exploratory qualitative research design. This design was identified as the most appropriate method to gain in-depth and rich experiences and perceptions of occupational therapists regarding factors affecting TBI rehabilitation, and to gain better insight into how occupational therapy service delivery can be strengthened in Gauteng, South Africa (Newton Suter 2012).

### Participant recruitment

Inclusion criteria included registered occupational therapists with three or more years of clinical experience and who were working in the field of adult neurorehabilitation in public healthcare facilities (e.g. clinics, hospitals and rehabilitation hospitals) in Gauteng, South Africa at the time of data collection. Purposive sampling was used to recruit participants working in specialist trauma units who admit the most TBI cases in the province. Thereafter, snowball sampling was used for further recruitment to locate occupational therapists working in mid-level hospitals and community clinics for the step-down and community reintegration of TBI patients. Transferability was ensured through variety in the sample, by inviting participants with differing demographic factors (Nowell et al. 2017) particularly variation in years of experience, level of qualification and healthcare setting.

### Data collection

The primary researcher and a research assistant facilitated four focus groups between April 2021 and June 2022. The first focus group was face-to-face, while the remaining three focus groups were conducted on an online platform, namely Microsoft Teams, because of coronavirus disease 2019 (COVID-19) restrictions. Each focus group was between 60 min and 90 min in duration and conducted in English; see Table 1 for demographic information. A focus group guide was used by the primary researcher to facilitate discussion among participants regarding the topic of enquiry, the barriers and facilitators affecting TBI rehabilitation, and strategies that can be used to strengthen TBI rehabilitation service delivery in Gauteng, South Africa. To ensure all participants contributed, the researcher posed a broad question to the group and used probing to explore the experiences and perceptions of participants in depth (Krueger & Casey, 2014). The research assistant managed group logistics demands, documented facial expressions and gave a summary at the end of the group discussion (Krueger & Casey 2014; Parker & Tritter 2006). Each group was audio-recorded. The audio files were transcribed verbatim by a skilled transcriber in preparation for data analysis. Transcriptions detailed each participant’s statements by participant code. These were sent to each participant for member checking to assist in the credibility of the study; two participants sent suggested minor changes (Birt et al. 2016).

**TABLE 1:** Demographics of research participants.

Participant code	Number of years and months of total working experience	Number of years and months experience in neurorehabilitation	Level of healthcare	Level of qualification	Focus group
P1	5 years 3 months	4 years 3 months	Regional	B OT	1 and 2
P2	5 years 4 months	4 years 6 months	Quaternary	BSc OT	1
P3	9 years	5 years	Quaternary	BSc OT	1
P4	3 years	2 years	Quaternary	BSc OT	1
P5	5 years 6 months	4 years and 6 months	District	BSc OT	1
P6	13 years	12 years	Tertiary	B OT	2
P7	12 years 6 months	10 years	Tertiary	MSc OT	2
P8	9 years 6 months	8 years 6 months	Quaternary	MSc OT	2
P9	21 years	10 years	Quaternary	MSc OT	2
P10	11 years 3 months	10 years 3 months	Tertiary	BSc OT	3
P11	15 years	14 years	Regional	BSc OT	3
P12	7 years 4 months	6 years 4 months	Tertiary	BSc OT	3
P13	3 years 6 months	2 years 6 months	Primary	BSc OT	4
P14	7 years 6 months	6 years 6 months	Primary	BSc OT	4
P15	10 years 6 months	9 years 6 months	Primary	BSc OT	4
P16	3 years 6 months	2 years 6 months	Primary	BSc OT	4

BSc, bachelor of science.

### Data analysis, trustworthiness and rigour of the study

Thematic data analysis was used for inductive data analysis (Parker & Tritter 2006) to allow themes and categories to emerge naturally from the data (Braun & Clarke 2021). The data were analysed through the six-phase process of thematic analysis (Braun & Clarke 2021). Manual coding and MAXQDA (Plus) were used to extract and develop a hierarchical structure of codes (Saillard 2011). Descriptive coding was used to summarise phrases or passages into topics. In vivo coding was used to preserve participants’ actual words (Saldana 2013). The same method of coding was repeated by the primary researcher 2 weeks later to ensure the dependability of the data (Morse 2015). Thereafter, a draft code manual and two focus group transcriptions were provided to two coders to manually code independently. The two independent coders were the research supervisor and a researcher familiar with thematic analysis in the same field of study. Discrepancies in coding between the primary researcher and independent coders were discussed to reach a consensus and clarify codes and code definitions (Morse 2015). Codes were then analysed by the primary researcher for patterns and connections to determine categories. Subsequently, categories were further refined by the primary researcher and research supervisor to finalise codes, categories and themes (Rossman & Rallis 2012).

Data triangulation was used to ensure credibility and confirmability of the study (Abdalla, Oliveira & Azevedo 2018). The triangulation technique used combined different sources and methods of data collection, namely group observations by the research assistant, reflexive notes by the primary researcher and the four group transcriptions. To ensure data saturation was met, a code frequency table was populated. Twenty-eight codes were generated in the first group, four new codes in the second group, two new codes in the third group and two new codes in the fourth group. Four groups were deemed sufficient because the two new codes that arose in the fourth group did not result in a new subcategory.

### Ethical considerations

Ethical approval to conduct the study was obtained from the Human Research Ethics Committee at a University in Johannesburg (reference number: M200958). Participants gave written consent to participate in the study. Confidentiality during the data analysis, results and publication that would arise from the study was ensured through participants being given a participant code (Krueger & Casey 2014). Participants were contacted via e-mail and made aware that they could withdraw from the study at any point. A non-disclosure agreement form was signed before the commencement of each focus group by each participant to reiterate confidentiality.

## Results

### Demographic information

A total of 16 occupational therapists participated in the study (see Table 1 for demographic information of participants). All participants were occupational therapists who were working in TBI rehabilitation in public health facilities in Gauteng, South Africa at the time of data collection. Most participants (56.25%) were working in acute specialist hospitals (tertiary and quaternary level care), fewer participants (18.75%) worked in mid-level hospitals (regional and district level care) and 25% worked in clinics (primary level care). The majority of participants (81.25%) held an undergraduate degree in occupational therapy, and the remainder (18.75%) held a master’s degree in occupational therapy. The range of working experience as a clinical occupational therapist was between 3 years and 21 years. The range of clinical work in the field of neurorehabilitation was between 2 years and 14 years (median: 6 years 5 months).

### Emerging themes

Three themes emerged which captured the essence of the barriers and facilitators to the provision of rehabilitation for patients with TBI in Gauteng public healthcare settings based on occupational therapists’ perspectives and experiences in TBI rehabilitation:

- ‘Not a recipe’: Alludes to the experience of TBI rehabilitation not having a set formula for assessment, intervention and general management: ‘It is not a recipe, and every patient is different.’ (P8)
- ‘Toolbox’: Encompasses all the people and processes involved in the TBI rehabilitation process. This includes all treatment, assessment and management of TBI: ‘… there is strategies sort of that you use from your … toolbox … that you use to address specific problems.’ (P8)
- ‘Lost in the cracks’: Reports on the perception of patients not receiving, completing or having successful rehabilitation: ‘Lost in the cracks.’ (P1)

All three themes encompassed both positive and negative factors that influenced TBI rehabilitation. The duality of the three themes is presented separately as the perceived barriers (Table 2) and facilitators (Table 3) to TBI rehabilitation by occupational therapists. Thereafter, strategies (Table 4) that were extracted from categories are presented.

**TABLE 2:** Barriers to traumatic brain injury rehabilitation.

Theme	Category	Direct participant quotes
‘Not a recipe’	Complexity	‘… you need to know not only about head injuries, but also about all the other injuries they might have sustained and the precautions around that.’ (P8, Focus group 2, Quaternary)
		‘the family does not really know what to do with them … they kind of just leave them in the bed and they come to us really stiff.’ (P12, Focus group 3, Tertiary)
		‘We have patients coming with severe fixed contractures … Perhaps they’re not being referred, perhaps therapists aren’t trained in managing a case like that.’ (P5, Focus group 1, District)
	Neurological impairments	‘You just don’t know what you are going to get. It could be a patient that presents with predominantly physical deficits, or it could be a person that they present with more cognitive perceptual deficits, or it could be a combination.’ (P14, Focus group 4, Primary)
		‘… your main focus initially starts with physical… then the cognition rehab gets neglected.’ (P7, Focus group 2, Tertiary)
		‘Patients getting back to work, managing in the home, returning to doing their previous occupations - the real hindrance usually is the cognition, and that is long lasting.’ (P8, Focus group 2, Quaternary)
		‘… return to work is usually impossible or not an option, and you have to look at putting them on grants soon.’ (P7, Focus group 2, Tertiary)
	Personal characteristics	‘… the problem with that is that they are young, and they should be part of our open labour market, they should be the contributing population, and so many of them can’t return to a normal work environment … they can’t support their dependents anymore.’ (P8, Focus group 2, Quaternary)
		‘… they come in as unknown males … you don’t get to get collateral information on what their premorbid functioning possibly is … they do sometimes stay a long time in hospital, or they need placement and sometimes these are foreigners as well.’ (P7, Focus group 2, Tertiary)
‘Toolbox’	Family	‘There is either family issues or their living situation is not great. So, the family support in that regard really is lacking.’ (P16, Focus group 4, Primary)
		‘Families do not understand how it is possible that … they might be mostly physically back to normal, but not cognitively and not in terms of their personality and their behaviour.’ (P1, Focus group 1 and 2, Regional)
		‘But how do you teach a family in like an hour, when you have to do family training and they will live with that patient?’ (P3, Focus group 1, Quaternary)
		‘… before that pass out, we would educate the family … [*you would*] be able to get feedback from the family in terms of what their challenges were and what went well.’ (P5, Focus group 1, District)
	Occupational therapist skills	‘… there is no protocol for TBI, because it is such a complex thing. But … you can have a set of handling principles.’ (P6, Focus group 2, Tertiary)
		‘We kind of have to fight for our patients to be admitted for a start.’ (P5, Focus group 1, District)
		‘Advocating for just having the patient being treated decently.’ (P1, Focus group 1 and 2, Regional)
	MDT influences	‘… our definition of functional maybe is different to what the doctors and the nurses see as functional … I had a TBI, and he was discharged … And I was like, but he can’t go home, because physically he was capable of doing x, y, and z but cognitively he couldn’t.’ (P3, Focus group 1, Quaternary)
		‘They’re not understanding the way that they need to explain to the patient or demonstrate or whichever instruction they’re giving. So, they’re either overload the patient or underload them.’ (P5, Focus group 1, District)
		‘… you have to stand up to say, okay let’s teach her how to dress independently, don’t quickly do the affected upper limb … (P9, Focus group 2, Quaternary)
	Educational influences	‘I feel like even university haven’t equipped us well enough to be with TBIs.’ (P4, Focus group 1, Quaternary)
		‘It’s possible that you can go into … the physical blocks and come back without seeing a TBI patient.’ (P4, Focus group 1, Quaternary)
		‘When you get training, the same things for so many aspects, it’s no longer fulfilling … it can get very frustrating.’ (P4, Focus group 1, Quaternary)
	Management influences	‘… it is also quite hard to establish the cluster system because I know our whole EXCO [*Executive Committee*] and management was trying to push from head office side to establish that clusters, but our central clusters are still not established yet.’ (P10, Focus group 3, Tertiary)
		‘The dissemination of information when it comes to … learnership programs that are out there. Sometimes our managements are also quite hesitant for us to get involved.’ (P11, Focus group 3, Regional)
	Documentation	‘… we can read the protocols and everything but those protocols, you find out most of them, they are not contextually relevant.’ (P4, Focus group 1, Quaternary)
		‘Cognition … isn’t as evidenced or … researched as much as physical rehab. So, we … have a lot more resources in terms of physical rehab.’ (P8, Focus group 2, Quaternary)
	Appropriateness	‘[*They*] wanting to do cognitive therapy, but are pitching it at the wrong level. So, they are expecting TBI patients to identify objects that are not familiar, or they don’t even attempt to communicate to them in their home language.’ (P7, Focus group 2, Tertiary)
‘Lost in the cracks’	Not seen until completion	‘… financial difficulties.’ (P13, Focus group 4, Primary)
		‘… stigma, depending on the mechanism of injury… They come for one or two appointments and that’s about it because the community doesn’t support them and neither does their family.’ (P14, Focus group 4, Primary)
		‘They don’t want to come for the appointments.’ (P14, Focus group 4, Primary)
		‘It has a lot to do with the cognitive functions or actually remembering the appointments and the insight.’ (P15, Focus group 4, Primary)
		‘Often I have gotten calls from therapists from other hospitals to ask if a patient has arrived at [X], most of the time the patient is not even at [X].’ (P11, Focus group 3, Regional)
		‘I do think the referrals are being done. It’s just that the patients are not coming or presenting to our departments with those referrals and usually when they are coming years later.’ (P14, Focus group 4, Primary)
	Rehabilitation package	‘It is harder to get these patients into rehab facilities … in terms of resources there is very little access to rehabilitation in Gauteng … because of those polytraumas and they often have a PEG tube, or an NG tube and the rehab facilities cannot cope with that or … they very aggressive, they have poor social circumstances, not good social support and rehab facilities do not [*accept these*] patients.’ (P8, Focus group 2, Quaternary)
		‘Usually at the clinics, we don’t see the Road Accident Fund patients as well as occupational injury patients, like Workmen’s Compensation patients.’ (P15, Focus group 4, Primary)
		‘… the community resources are extremely low.’ (P5, Focus group 1, Quaternary)
		‘I usually refer my patients to [A], alongside my therapy so that they do get the counselling and the support and everything else that they need for the rest of the time that I’m not seeing them.’ (P14, Focus group 4, Primary)
		‘There’s also two sheltered workshops in [B] and another one in [C], but they are always full so they also do have long waiting lists so it can take a while for the patients to be accepted.’ (P16, Focus group 4, Primary)

*Source:* Africa, L.E., Human, A. & Tshabalala, M.D., 2023, ‘Participation patterns of children with cerebral palsy: A caregiver’s perspective’, *African Journal of Disability* 12(0), a1058. https://doi.org/10.4102/ajod.v12i0.1058

TBI, traumatic brain injury; MDT, multidisciplinary team.

**TABLE 3:** Facilitators to traumatic brain injury rehabilitation.

Theme	Category	Direct participant quotes
‘Not a recipe’	Personal characteristics	‘… with our young TBI’s, it bodes well for them in terms of recovery because … neuroplasticity … that is a positive for them because they can recover more … and that pre-morbidly they are quite healthy.’ (P8, Focus group 2, Quaternary)
‘Toolbox’	Family	‘… sometimes getting a family member, just snaps those patients out of like their low level of arousal and they participate so much better.’ (P2, Focus group 1, Quaternary)
		‘… caregiver training is probably the largest part of our job.’ (P13, Focus group 4, Primary)
	Occupational therapist skills	‘A lot of handling and structure, and then using activities that are meaningful for the patient, that’s what I find helps the most.’ (P8, Focus group 2, Quaternary)
		‘Your best course of action is just to work your way up, your creative ability levels.’ (P6, Focus group 2, Tertiary)
		‘… with the Ranchos Los Amigos scale because their behaviour changes you can see their levels of creative ability change quite easily … other normal neuro assessments like our modified Ashworth for tone, and Brunnström stages to see their motor control.’ (P8, Focus group 2, Quaternary)
	MDT influences	‘I think emphasis comes down to having a good MDT and just understanding their scope and what needs to be done and communication amongst the team just to coordinate those efforts.’ (P10, Focus group 3, Tertiary)
	Educational influences	‘We need to … problem solve a bit more because that’s ultimately what you have to do as a therapist.’ (P3, Focus group 1, Quaternary)
	Appropriateness	‘Basic ADL’s … giving a patient a basin with some water and soap and asking him to wash his face or put cream will tell you a lot.’ (P7, Focus group 2, Tertiary)

*Source:* Africa, L.E., Human, A. & Tshabalala, M.D., 2023, ‘Participation patterns of children with cerebral palsy: A caregiver’s perspective’, *African Journal of Disability* 12(0), a1058. https://doi.org/10.4102/ajod.v12i0.1058

TBI, traumatic brain injury; MDT, multidisciplinary team.

**TABLE 4:** Strategies to improve traumatic brain injury rehabilitation.

Theme	Category	Direct participant quotes
‘Toolbox’	Educational influences	‘I think it is just the lack of workshops sort of geared towards TBI … just to add to try and develop these services.’ (P10, Focus group 3, Tertiary)
		‘… a bit more training around treating TBIs and I think it’s something we might have to make specific for community because there aren’t resources for that.’ (P16, Focus group 4, Primary)
	Documentation	‘… there is actually no emphasis based on TBI work groups … we can maybe try and solve that problem in terms of establishing … better TBI protocols.’ (P10, Focus group 3, Tertiary)
	Appropriateness	‘… if we can’t communicate with the patients, then we also need to start bringing in our families more. They can communicate with the patients …’ (P2, Focus group 1, Quaternary)
‘Lost in the cracks’	Not seen until completion	‘Health promotion and prevention around TBI will actually help a lot with acceptance into the community.’ (P15, Focus group 4, Primary)
	Rehabilitation package	‘If we had more access to rehabilitation facilities for patients that are confused and have polytrauma. So, I think in easing … the admission criteria to get into rehabilitations.’ (P8, Focus group 2, Quaternary)
		‘… a resource document of sorts that could be sent to each hospital of these kinds of services.’ (P12, Focus group 3, Tertiary)

*Source:* Africa, L.E., Human, A. & Tshabalala, M.D., 2023, ‘Participation patterns of children with cerebral palsy: A caregiver’s perspective’, *African Journal of Disability* 12(0), a1058. https://doi.org/10.4102/ajod.v12i0.1058

TBI, traumatic brain injury.

### Barriers to traumatic brain injury rehabilitation

#### Theme 1: ‘Not a recipe’

In the category ‘complexity’, occupational therapists experienced TBI rehabilitation as a difficult and complex process. Participants highlighted the variation among patients with TBI with regard to severity, and overall clinical presentation. Contributing to the complexity of TBI rehabilitation is the occurrence of polytrauma and other complications. Participants based in hospital settings (i.e. quaternary, tertiary, regional and district levels of healthcare) explained that good knowledge and skill are needed to manage the polytraumas these patients experience. Concern regarding the management of additional complications by all multidisciplinary members, as well as family members once they are discharged, was voiced.

In the ‘neurological impairments’ category, participants from all levels of healthcare expressed the unpredictability of the various long-term sequelae of TBI. These impairments include a combination of cognitive, physical and perceptual deficits. One participant explained that because of the various neurological impairments, physical recovery is often prioritised over cognitive rehabilitation. Another participant emphasised that the cognitive, behavioural and emotional impairments were the most long-lasting. Furthermore, participants reported that the cognitive and behavioural deficits most greatly impact a patient with TBI’s ability to return to work and reintegrate back into their home and society. As a result of these deficits, occupational therapists perceive return to work as ‘impossible’ and suggested that TBI clients be referred for government disability grant schemes for financial support.

The category ‘personal characteristics’ of the patients further add to the barriers of TBI rehabilitation. Patients with TBI were reported to be predominantly young adult men of working age who should be at the peak of contributing to the economy and a financial support to their families. Another personal characteristic that adds to the barriers of rehabilitation is that patients with TBI are frequently unknown (i.e. no identification) or foreigners and have no known familial support and collateral information cannot be obtained contributing to a long hospital stay.

#### Theme 2: ‘Toolbox’

In the ‘family’ category, participants in all levels of healthcare frequently discussed the family support for patients with TBI during the rehabilitation process being a tool that can hinder recovery. Patients with TBI often have pre-existing social issues that complicate TBI management and little familial acceptance and support during the rehabilitation process can hamper a patient’s recovery. The impact that various neurological impairments have on the family structure was highlighted by participants. The family typically goes through a grieving process of mourning the loss of who that person was prior to the TBI incident. The lack of understanding of the effects of a TBI by family members influences insight. The participants further reported how detrimental it can be to the patients if they are discharged without adequate education and support for family on how to handle the TBI survivor. In addition, a participant highlighted how COVID-19 minimised the opportunity for caregiver training and weekend pass-outs, which could benefit the rehabilitation process.

In the ‘occupational therapists skills’ category, emphasis was placed on treatment interventions, assessments and management that they perceived as valuable or requiring improvement with relation to TBI rehabilitation. Some participants highlighted the need for a set guidelines on handling principles for these patients, which is currently lacking. Advocacy was perceived as another occupational therapy skill in the ‘Toolbox’. Advocacy was perceived to be required for simply getting patients with TBI admitted for rehabilitation or to be handled in a fair manner by participants in the acute levels of healthcare.

In the ‘MDT influences’ category, participants perceived themselves to carry out TBI rehabilitation more holistically than their other MDT members. When not done, this was perceived to hinder patients’ progress, as there is limited understanding on the multifaceted presentation of these patients, which can affect their rehabilitation outcomes. Concerns about the way patients with TBI are handled and how to pitch and structure their sessions were further highlighted. The participants in hospital-based settings reported the need for MDT members to understand the value of allowing the patient to engage in activities as independently as possible.

The ‘educational influences’ category highlighted the level of training provided in TBI to occupational therapists, and the amount of experience and exposure to patients with TBI was another major point of discussion. Participants from all levels of healthcare felt that the undergraduate occupational therapy programme needs to be re-evaluated to better equip occupational therapists to engage with patients with TBI. A lack of experience and training is particularly highlighted with community service and junior occupational therapists. Feelings of frustration were shared with regard to having to teach a continuously changing cycle of novice therapists the same concepts on TBI rehabilitation.

In the ‘management influences’ category, it was highlighted that negative practices by higher management, such as poor dissemination of information, can negatively impact TBI rehabilitation.

The ‘documentation’ category related to participants reported that TBI protocols were not contextually relevant. In addition, there is a lack of research on cognition with regard to neurological conditions, and this subsequently affects the quality of rehabilitation.

In the ‘appropriateness’ category, three aspects were highlighted as key factors that could negatively affect a therapeutic session. These were the language used to speak to the patient, the just-right challenge and the contextual relevance the intervention provided.

#### Theme 3: ‘Lost in the cracks’

In the category ‘not seen until completion’, many primary healthcare participants experienced patients defaulting as outpatients which affects patients with TBI not being seen until completion of the rehabilitation process. Aspects including substance use, poor socio-economic backgrounds, a lack of finances, poor familial support, community stigma, a lack of insight and motivation, and poor cognitive functioning are contributing factors to defaulting. The referral system was discussed as another factor contributing to a patient getting lost in the system.

The ‘rehabilitation package’ category describes the lack of access patients have to all TBI rehabilitation services. Participants in acute levels of healthcare (i.e. quaternary) reported that many patients with TBI do not qualify for admission to rehabilitation hospitals. Admission criteria to rehabilitation hospitals include, but are not limited to, patients having a PEG tube, poor social support or aggressive behaviour. This barrier further aggravates patients getting lost in the system. A participant in primary healthcare explained that patients with a TBI because of a motor or pedestrian-vehicle accident or an injury on duty, do not have access to primary healthcare services at clinics because of billing systems with the Road Accident Fund and Worker’s Compensation. Participants from all levels of healthcare reported a lack of community resources for patients with TBI and their family members. In addition, community resources available for vocational opportunities to allow the return to work for patients with TBI are limited.

### Facilitators to traumatic brain injury rehabilitation

#### Theme 1: ‘Not a set recipe’

Under ‘personal characteristics’, it was noted that young patients often have a better premorbid health status than older patients and neuroplasticity – the key to neurorehabilitation – is more effective in the young brain to allow for good functional outcomes.

#### Theme 2: ‘Toolbox’

In the ‘family’ category, it was reported that if patients with TBI have good familial acceptance and support during the rehabilitation process, this will act as a facilitator in their recovery. The participants in all levels of healthcare, particularly in primary healthcare, expressed the importance of caregiver training in therapy.

The ‘occupational therapists skills’ category encompasses the treatment interventions, assessments and management of patients with TBI by occupational therapists. Participants reported that skilled intervention planning in occupational therapy (client-centred activity selection, structuring a therapeutic activity and correct manual handling of the patient) is essential for successful outcomes. Participants with work experience of 8 years or more or had their master’s degree in occupational therapy reported on more specific frames of reference, models and assessments to guide their intervention with patients with TBI. These participants reported the positive use of the Model of Creative Ability to guide overall intervention for these patients. The Ranchos Los Amigos scale, Brunnström approach and other physical assessments were further reported as useful assessment measures during TBI rehabilitation.

The ‘MDT influences’ category reflects how participants experienced a skilled MDT and effective communication among the MDT as a facilitator to TBI rehabilitation.

In the category ‘educational influences’, the majority of participants reported the need for more emphasis on TBI on an undergraduate level. One participant based in a quaternary level hospital, highlighted the need to use one’s own clinical reasoning to problem solve when treating patients with TBI. Using one’s own clinical reasoning was perceived as helpful as TBI presentation is very broad and a set intervention strategy for these patients may not always be a possibility.

Under the ‘appropriateness’ category, participants reported the value of using activities to provide contextually relevant intervention for their patients with TBI. Participants further reported the Model of Creative Ability as a good tool to allow for the just right challenge for patients with TBI.

### Strategies to improve traumatic brain injury rehabilitation

#### Theme 2: ‘Toolbox’

Under ‘educational influences’, improved training opportunities in the form of workshops or courses were suggested to be a valuable strategy for improved TBI rehabilitation by participants in all levels of healthcare.

Under the ‘documentation’ category, participants proposed the development of TBI protocols which are contextually relevant to improve TBI intervention. A possible facilitator to protocol development is the formation of a provincial workgroup as suggested by one participant. Another participant in an academic quaternary level hospital reported the importance of more research targeted at cognition to improve TBI rehabilitation.

The ‘appropriateness’ category included participants’ experience of providing therapeutic intervention in the patient’s home language, and the need to use family members to communicate was reported as a solution to providing therapy in the patient’s home language.

#### Theme 3: ‘Lost in the cracks’

In the ‘not seen until completion’ category, a potential strategy mentioned by one of the participants, in primary healthcare, was that more prevention and promotion measures need to be put in place to manage the stigma of TBI in communities that results in defaulting treatment.

In the ‘rehabilitation package’ category, one participant in a quaternary level hospital reported that there is a need to ease admission criteria at rehabilitation facilities to accommodate TBI patients with polytrauma or cognitive impairments. It was suggested by a participant in tertiary level of healthcare that a resource document should be made on the various training opportunities available for these patients for easier referral and management.

Strategies related to improving the intervention provided to patients with TBI included improved communication, improved education and training on TBI, protocol development and targeted research. Furthermore, strategies to minimise defaulting and improve accessibility to services at all levels of healthcare are valuable.

## Discussion

### Barriers to traumatic brain injury rehabilitation

In this study, occupational therapists perceived TBI rehabilitation as complex, owing to the range in severity and the unpredictable clinical presentation of TBIs. Occupational therapists found this negatively impacted their rehabilitation planning and implementation. Furthermore, polytrauma and complications have been found to increase the complexity of the TBI rehabilitation process (Capizzi, Woo & Verduzco-Gutierrez 2020; Webster, Taylor & Balchin 2015). Polytrauma is common in TBI cases because the mechanism of injury – typically violence (e.g. assault) or injury (e.g. road accidents) – often presents with other injuries, such as fractures (Yue et al. 2020). Cannoy (2021) found that occupational therapists in the United States of America are expected to use not only neurologically based frames of references to manage patients with TBI but also a more biomechanical approach to treatment to address these polytraumas. Similarly, in this study, participants reported that TBI rehabilitation requires a broad knowledge base of the management of various diagnoses.

Polytraumas and complications were not reported by primary healthcare (clinic) occupational therapist participants, because of this being a potentially more common concern initially at hospitals.

Patients with TBI can present with an array of physical, cognitive, behavioural, emotional and perceptual dysfunctions (Rabinowitz & Levin 2014; Webster et al. 2015), all of which were highlighted in the various participant discussions. With this complexity, occupational therapists are expected to have advanced clinical reasoning abilities, as no patient with TBI is the same.

Therefore, occupational therapists are expected to integrate multi-faceted deficits to ensure appropriate treatment plans are put in place for these patients.

Cognitive, emotional and behavioural deficits were perceived to be the most prominent persistent impairment in TBI survivors by occupational therapists in this study. This finding is supported by the reported 65% prevalence of long-term cognitive difficulties experienced by moderate to severe TBI cases (Rabinowitz & Levin 2014), as well as behavioural and emotional impairments being present in 60% of cases (Trevena & Cameron 2011). In contrast, only 30% of TBI cases have a persistent physical disability (Berger et al. 1999). Despite the greater burden of cognitive deficits long-term, participants revealed that in TBI management, physical rehabilitation is favoured over cognitive rehabilitation in occupational therapy. Participants further explained that cognition was found to be less understood by occupational therapists and not as greatly researched in comparison to physical deficits. This is supported by past studies that have shown that cognitive, behavioural and emotional impairments are often omitted and misunderstood (Andrew, Rothemeyer & Balchin 2017; Berger et al. 1999; Wilson 2017).

Impairments influence a person with TBI’s ability to successfully re-integrate back into the home and community, as seen in previous studies (Camp, Casteleijn & Thupae 2020; Capizzi et al. 2020; Webster et al. 2015). Furthermore, the cognitive, emotional and behavioural impairments post-TBI impact on an individual’s ability to return to work (Andrew et al. 2017; Khan, Baguley & Cameron 2003). These findings support the current study, where numerous participants reported poor integration back into the home, community and workspaces post-TBI because of cognitive and behavioural deficits.

In this study, participants viewed TBI occurrence in this demographic to have financial implications, not only to the families of patients with TBI but also to the economy as a whole. Wong et al. (2016) highlighted that TBI is most prevalent in 15–34-year-old males in Africa, a substantial demographic in a country’s workforce. Occupational therapists play a key role in return-to-work assessment and management following a disability (Birkhead 2014). However, in this study, occupational therapists experienced poor vocational reintegration of clients with TBI. This finding may only reflect the experiences of the study sample; participants were all working in public healthcare in South Africa and serving the nation’s poorest people in the country that has one of the highest unemployment rates in the world at 32.9% (Statistics South Africa 2023). Occupational therapists in this study referred patients for government disability grants to mitigate the financial strain on families but arguably placing a long-term burden on the local economy.

Occupational therapists expressed difficulties in navigating the assessment of clients with an unknown identification (i.e. no known identification or contacts). Occupational therapists experienced difficulties in gaining meaningful background information from an unknown patient, a key component in the development of an occupational profile of an individual (Hansell, Bissett & Caine 2022).

Therapists mentioned that some of their unknown patients are foreigners too, adding further language, financial and legal barriers to accessing rehabilitation services. South Africa hosts the highest number of immigrants on the African continent and foreigners in South Africa often have difficulty gaining access to medical services (Landau, Ramjathan-Keogh & Singh 2005). With various barriers to appropriately assess patients with a TBI, this results in the lack of a client-centred and holistic approach.

Perlesz, Kinsella and Crowe (2000) found that 49% of family members go through psychological distress following TBI. This was also observed by participants who emphasised the vast impact TBI has on patient’s support systems. A South African study reports how families often do not feel adequately educated on TBI and its potential effects (Webster et al. 2015). In addition, families are not provided with adequate coping strategies to manage the TBI consequences (Webster et al. 2015), with significant impacts on interpersonal relationships within the family dynamic (Wedcliffe & Ross 2001). In this study, occupational therapists echoed these concerns and felt that time provided to train caregivers is inadequate. During COVID-19 restrictions, family support was further impeded by preventing patients from weekend home visits – these ‘pass-outs’ were perceived to be important opportunities for occupational therapists to receive feedback on home reintegration during the rehabilitation process.

Many young occupational therapists feel unprepared when faced with a patient with TBI because of a lack of experience and exposure, which results in limited clinical reasoning skills when dealing with this patient population, which necessitates the need for improved training in TBI rehabilitation (Ned et al. 2020). Furthermore, more experienced occupational therapists are in private practice or academia, leaving younger, inexperienced therapists to manage complex TBI cases in public facilities (Ned et al. 2020). In South Africa, the *Health Professions Amendment Act No. 56* enacted new graduates to complete a compulsory community service year to serve in public health facilities. Although this fills a much-needed gap in service delivery, the annual staff turnover results in a stream of new therapists that require further training in the management of complex conditions in specialised hospitals. This study found that skilled occupational therapists in hospital settings felt frustrated by the need to continuously teach a new cycle of junior therapists.

In this study, occupational therapists expressed frustration between their assessment of when a client with TBI is ready for discharge, compared to a nurse or doctor’s perspective. This contrast may be owing to different theoretical frameworks: on one hand, many doctors within the rehabilitation team for patients with TBI using a biomedical model (Kusnanto, Agustian & Hilmanto 2018) which focuses on treating the body, whereas occupational therapists are concerned with all factors influencing engagement in occupations. Interprofessional discharge planning meetings (including the patient and caregiver) to improve communication among the team should be prioritised (Lutz et al. 2022). In addition, participants also felt that higher management further plays a role in the rehabilitation of patients with TBI, as they often can assert authority and make decisions on the overall management of patients with TBI.

There are several international guidelines for TBI; however, they are not contextually relevant to our unique circumstances (Pitama et al. 2017). There are no current TBI rehabilitation guidelines that have been developed for the South African setting. This study highlighted that occupational therapists do not feel that there are appropriate guidelines to assist in the management of patients with TBI.

There is a strong need for improved research, particularly in cognitive rehabilitation, to strengthen service delivery for these patients. This study further highlights the overall limited research that has been done with regard to TBI rehabilitation in South Africa (Soeker, Van Rensburg & Travill 2012).

The language in which the session is conducted further influences the level of success a patient has during therapeutic intervention (Watt, Penn & Jones 1996). This can impact the therapeutic relationship with the patient and subsequently influence their engagement in rehabilitation. Language is a necessary tool to gain valuable information from the patient to ensure client-centred management. Language barriers, cognitive deficits and the impact of unknown patients all influence the amount of valuable information we need to obtain on these patients. Although, experienced occupational therapists can generalise from past experiences and patient’s context to make clinical reasoning decisions for the rehabilitation of these patients, despite having limited information. Whereas those occupational therapists with limited experience do not have any frame of reference when treating these patients, making their treatment planning difficult.

In this study, loss to follow-up of patients with TBI was a common barrier experienced among occupational therapists, particularly in primary healthcare settings. Participants further explained that patients with TBI are predominantly from poor socio-economic backgrounds with limited finances and resources to adhere to the rehabilitation process once they are discharged home (Andrew et al. 2017; Joosub 2019; Maasdorp, Swanepoel & Gunter 2020; Naidoo 2013). Substance abuse and poor socio-economic circumstances appear to be some of the predisposing factors leading to TBI incidences, as reported in literature (Joosub 2019; Tipton-Burton, McLaughlin & Englander 2013) and are further emphasised in the current study. In addition, occupational therapists experienced a loss to follow-up for outpatient appointments where there was low acceptance of the TBI survivor by community members and family (Jeyaraj et al. 2013). Cognitive impairments including poor insight and lack of motivation, further result in TBI survivors not returning for appointments (Andrew et al. 2017; Bainbridge 2015; Barman, Chatterjee & Bhide 2016; Capizzi et al. 2020; Webster et al. 2015), as found in this study. These factors contribute to a premature end to a TBI survivor’s rehabilitation journey, hindering their potential for further recovery.

International studies recommend inpatients with TBI should receive occupational therapy at least five times a week or alternatively be sent to a step-down facility if not suitable for inpatient rehabilitation (Capizzi et al. 2020; Chua et al. 2007). It is further recommended that outpatient therapy should continue for at least 1–2 years post-TBI (Capizzi et al. 2020; Chua et al. 2007). From this study, it is evident that this is not occurring in occupational therapy in the public healthcare facilities in Gauteng. Patients are receiving limited rehabilitation and participants expressed that few patients with TBI are admitted to rehabilitation facilities because of admission conditions. The participants in secondary, tertiary and quaternary levels of healthcare settings experience the need to advocate for patients to be admitted to rehabilitation facilities and perceived the admission criteria as a barrier to TBI rehabilitation. Participants further explained that rehabilitation hospital admission criteria exclude patients with common impairments seen in TBI such as confusion and felt these restrictions needed to be eased. Lastly, participants experienced the referral of patients with TBI from one facility to another, and referrals between levels of healthcare, was problematic. Participants felt that this contributed to patients getting lost in the system and not receiving essential healthcare services, as they cannot effectively follow-up (Andrew et al. 2017).

Previous studies have reported that there is a lack of outpatient therapy services available in South Africa (Capizzi et al. 2020). This was reiterated in this study by participants in all levels of healthcare, where it was found that billing systems with the Road Accident Fund and Workers Compensation Fund are managed only at a hospital level and not by primary healthcare (Korbin 2014; South African Government 1993). Patients with TBI are therefore not always able to receive primary healthcare services in the form of home visits and essential support services. This places further financial strain on the patient and their families, as they are expected to travel further to hospitals, as opposed to receiving healthcare services at their local clinic.

A study conducted in Western Cape found limited support groups and facilities which offer continuous cognitive rehabilitation services for patients with TBI (Wilson et al. 2015). There were similar findings in this study, where there are limited community resources in the form of outpatient support groups for both the family and patients, as well as limited vocational opportunities available for patients with TBI. There is an urgent need for more support groups in various districts of the province, expressed by participants in all levels of healthcare. Many occupational therapists are not aware of what vocational opportunities are available in Gauteng and there is insufficient space in these facilities to accommodate the large number of patients with TBI who require it.

### Facilitators to traumatic brain injury rehabilitation

Although TBI rehabilitation is viewed as complex and multifaceted by occupational therapists, some factors may be promising facilitators to recovery. Participants viewed age as a two-sided coin; TBI in young adults was discussed to have a negative impact on the worker role but was also viewed as a good prognostic indicator for recovery. This perception is possibly because of the knowledge that neuroplasticity occurs more effectively in the young brain (Kleim & Jones 2008). In addition, participants experienced young TBI patients to have good premorbid health, lending to a more favourable outcome in rehabilitation (O’Donnell et al. 2010). The unique combination of individual factors that constitute a patient’s prognosis – such as age – highlights the notion that rehabilitation is different for every individual.

Both national and international studies found that education and support for family members from as early as the acute stages of care is imperative (Birkhead 2014; Broodryk & Pretorius 2015; Lam Wai Shun et al. 2017). This was reiterated in this study, where it was found that successful family training and education allows for improved family support during the rehabilitation process, allowing for better functional outcomes for these patients. Thus, education must be done in a timely manner, and be relevant, practical and understandable for the family member.

Education is not only a necessity with family members but also with other multi-disciplinary team members on how to manage patients with TBI effectively, which maximises their rehabilitation process.

Although many of the participants felt that there were no guidelines tailored to the South African population, the Model of Creative Ability has been found, in both previous literature and this study, to be a useful model during TBI rehabilitation to provide treatment principles regarding activity selection, structuring of a therapeutic activity and presentation thereof (Birkhead 2014).

The Ranchos Los Amigos scale is also valuable as a tool to understand the behavioural and cognitive presentation of a patient with TBI (Lin & Wroten 2022). The Modified Ashworth scale is further useful to assess hypertonicity in brain injuries (Mehrholz et al. 2005). Brunnström approach recovery stages have been found to be beneficial in determining upper limb function post neurological insult (Naghdi et al. 2010). The use of Vona du Toit’s Model of Creative Ability, the Ranchos Los Amigos scale, Modified Ashworth Scale and Brunnström stages of motor recovery were perceived by participants in hospital settings as valuable tools to use in their intervention and evaluation of patients with TBI. These tools may be more useful in the acute stage of recovery, a possible reason why primary healthcare participants did not discuss them. Primary healthcare participants emphasised the need to work on the goals reached together with the caregivers, maintenance of function and compensatory strategies to improve independence. The contrast between the focus on body functions and structures in the acute stage (e.g. Brunnström stages of motor recovery) compared to the focus on engagement in activities and participation in the chronic stage requires further investigation. Furthermore, the validity of most of these tools in LMICs remains to be investigated.

An MDT approach to TBI rehabilitation is found to be essential for effective and quality intervention for these patients (Barman et al. 2016; Camp 2015). The results show this through discussions on the value of an experienced and well-trained MDT in TBI intervention. Having good communication between MDT members further facilitates holistic rehabilitation for patients with TBI.

Research shows that occupational therapists in South Africa have difficulty treating patients with TBI once they have completed their undergraduate programme (Freeme 2011). However, it was noted in this study that participants who expressed that they utilised their own clinical reasoning skills felt more confident treating patients with TBI. In addition, participants experienced the use of everyday life activities that are familiar to a patient with TBI as a facilitator in their assessment and treatment. It is through these meaningful activities that a patient’s quality of life will be enhanced (Huebner et al. 2003).

### Strategies to improve traumatic brain injury rehabilitation

Translation has been highlighted as a potential strategy to allow better communication between healthcare providers and patients (Morris et al. 2021). The study provided the strategy of using the patient’s family members themselves to assist in communication.

The participants in all levels of healthcare reported the need for an undergraduate curriculum which targets the specific requirements and training to deal with patients with TBI in a practical setting, with adequate theory to substantiate one’s clinical reasoning, which is supported by a South African study (Freeme 2011). Courses and workshops targeted at TBI rehabilitation were suggested by participants as strategies to allow for continued professional development in this area and to equip them in the management of TBI.

As a result of limited guidelines that are contextually relevant, participants report the need for protocols and guidelines for TBI rehabilitation which are catered to the South African context (Pitama et al. 2017). In a country such as South Africa where there are diverse cultures and limited resources, it is important that guidelines used for neurorehabilitation are tailored to the specific context (Pitama et al. 2017). A provincial workgroup specifically for TBI can be established to assist in further contextually relevant protocols. Furthermore, the study highlighted the need for more research in the area of cognition to improve TBI rehabilitation (Andrew et al. 2017; Berger et al. 1999; Wilson 2017).

It is essential to put prevention and promotion programmes in place to better educate family and community members on the needs of patients with TBI to minimise stigma and allow increased support (Jeyaraj et al. 2013). This was a strategy suggested by a participant in primary healthcare; however, this strategy should extend to other levels of healthcare to allow for adequate implementation of the National Health Insurance goals (*South African Government: National Health Act* 2003) (South African Government 2015). This was emphasised in the current study, as there is often stigma surrounding patients with TBI.

The need for rehabilitation facilities that cater to patients with TBI’s needs is of great necessity. This includes facilities that have adequate training in managing of TBI patients and their various complexities.

Previous studies have reported the necessity of incorporating vocational intervention as a successful part of TBI rehabilitation (Birkhead 2014; Khan et al. 2003). This study emphasises the need for more attainable vocational opportunities for patients with TBI, particularly for the largely young TBI population of Gauteng. Productive occupations such as work will help mitigate the social and economic burden of disability and restore age and gender roles in individuals with TBI. Furthermore, it was suggested that a resource document is developed to make occupational therapists listing the vocational opportunities that exist in their community to prevent patients falling through the cracks.

### Limitations

As a result of COVID-19 restrictions, as well as for easing focus group logistics, only the first focus group was face-to-face, while the remaining three focus groups were conducted on an online platform: Microsoft Teams. The online focus group discussions could not document non-verbal communication which would have added increased depth to the study (Krueger & Casey 2014). In the first group, it was found that there was mostly consensus in the group observed through nodding and affirmative sounds, likely because of the homogenous sample. The fourth focus group comprised of only primary healthcare occupational therapists, making the discussion lack the necessary depth, as there were no participants from other levels of healthcare to share different or similar experiences. A fifth focus group of return participants from all levels of healthcare was anticipated to allow for a heterogenous group to discuss varied opinions; however, there was a poor response rate for a return group. This made valuable potential comparisons between participants in various levels of healthcare difficult to determine. The first focus group consisted of five participants, the second comprised of four participants, while the third and fourth focus groups comprised three and four participants respectively. Based on Kielhofner’s (2006) recommendations, it is suggested that five to six participants are involved in each focus group; however, because of a lack of availability of participants, most groups were made up of less than five participants. Lastly, connectivity issues disrupted group discussions at times.

## Conclusion

The aim of this study was to explore barriers and facilitators to the provision of TBI rehabilitation from occupational therapists’ perspectives within public healthcare settings in Gauteng, while determining strategies to improve service delivery. It is evident from the results that although there are factors which support and enhance service delivery for patients with TBI rehabilitation, there are more factors impacting the quality of rehabilitation for these patients. Strategies to overcome barriers to TBI rehabilitation include making use of translators, revision of the occupational therapy undergraduate curricula in South Africa, as well as the development of workshops and courses on TBI management. The development of provincial workgroups for TBI, improved research on cognition in TBI and the development of contextually relevant guidelines, local policies and protocols pertaining to TBI are also required. Finally, TBI prevention and promotion strategies need to be put in place, improved admission of patients with TBI to rehabilitation facilities, as well as the development and improved accessibility to community resources (both support groups and vocational opportunities) are further strategies that can be explored.

### Recommendations for future research

This study only explored the perceptions of TBI rehabilitation with occupational therapists in the Gauteng region of South Africa. Research in other provinces of the country will allow comparisons to be made regarding the barriers and facilitators to TBI rehabilitation across South Africa. This will allow a better understanding on a larger scale, while potentially providing more valuable insight into the rehabilitation process of these patients. Exploring the perspectives of TBI rehabilitation with other MDT members who are involved within the TBI rehabilitation process will provide more holistic viewpoints on the barriers and facilitators to TBI rehabilitation, while providing a different lens on various strategies to overcome the barriers experienced. It is further recommended that the barriers of TBI be explored through a WHO International Classification of Functioning, Disability and Health Environmental Factors framework.
